# Green Synthesis of Nanostructure CeO_2_ Using Tea Extract: Characterization and Adsorption of Dye from Aqueous Phase

**DOI:** 10.1155/2021/5285625

**Published:** 2021-12-15

**Authors:** Chengshun Liu, Xiyao Liu, Yilin Wu, Zhuotong Chen, Zhuanrong Wu, Shumao Wang, Hua Han, Zhenbang Xie, Yixuan Wang, Tzu-Hsing Ko

**Affiliations:** ^1^Fujian Provincial University Key Laboratory of Green Energy and Environment Catalysts, College of Chemistry and Materials, Ningde Normal University, Ningde, Fujian 352100, China; ^2^South China Botanical Garden, Chinese Academy of Sciences, Guangzhou, Guangdong, China; ^3^University of Chinese Academy of Sciences, Beijing, China; ^4^College of Horticulture and Forestry Sciences, Huazhong Agriculture University, Wuhan, Hubei, China; ^5^Institute of Tea Science, Zhejiang University, Hangzhou, Zhejiang, China

## Abstract

Nanostructure CeO_2_ powders were synthesized using tea waste extract as gel precursor. The as-prepared samples were characterized by thermogravimetric analyzer (TGA), X-ray diffraction (XRD), scanning electron microscopy (SEM), X-ray photoelectron spectroscopy (XPS), and Raman spectroscopy. Based on the TGA/DTG analysis, the intermediates of cerium chloride hydrates (CeCl_3_.4H_2_O and CeCl_3_.H_2_O) and cerium anhydrous (CeCl_3_) were produced, and the formation temperature of CeO_2_ was estimated to be 773 K. The cubic fluorite structure of CeO_2_ was detected to be the predominant species and was completely formed at the calcination temperature of 773K–1073 K with a crystal size between 8.8 and 11.4 nm based on the XRD measurement. Moreover, the main chemical state of ceria on the surface of the synthesized samples was confirmed to be tetravalent ceria by XPS. All samples show a strong Raman signal at a well-defined chemical shift of 463 cm^−1^ and a significant symmetry feature was observed, suggesting that the tetravalent ceria is the dominant species throughout the bulk sample. All the synthesized CeO_2_ calcined at different temperatures showed higher adsorption efficiency for Congo red (CR) compared with commercial CeO_2_. The adsorption efficiency maintained a steady state of more than 95% when the concentration of CR and adsorption temperature were varied in this study. The kinetic analysis showed that the second-order model was the appropriate model to interpret the adsorption behavior of synthesized CeO_2_. The calculated adsorption capacity derived from the second-order model is in good agreement with the experimental data. The isotherm analysis revealed that the Freundlich and D-R models fit well for the synthesized CeO_2_ and represent physisorption with a multilayer mechanism. The thermodynamic parameters, including the changes in Gibb's free energy, enthalpy, and entropy, suggested that the adsorption of CR on the synthesized CeO_2_ sample was a spontaneous and endothermic process.

## 1. Introduction

In recent decades, rare earth oxides have attracted the most attention because of their unfilled 4f electron structure, leading to extensive use in many fields. Among the rare earth oxides mentioned in industrial applications, cerium dioxide has received enormous attention because it can be used as luminescent materials [1], solid oxide fuel cells [2], and catalyst [3] and amperometric oxygen monitors [4]. It is undeniable that the synthesis, preparation, and application of CeO_2_ will continue to be a popular topic in the future. Current methods for the synthesis of CeO_2_ include chemical precipitation [4–6], hydrothermal process [7], sol-gel process [8], spray pyrolysis [9], microemulsion method [10], and electrochemical process [11]. Although these techniques are widely used for the synthesis of CeO_2_, they are complex, time-consuming, and involve a larger amount of hazardous chemicals, which drive up production costs and pose a serious threat to the environment and ecosystem. Moreover, they are inefficient due to higher cost, tedious downstream processing, lower biocompatibility, instability, and low yield [12–14]. Therefore, it is necessary to develop a rapid, simple, inexpensive, and environmentally friendly synthesis method for CeO_2_. Currently, many efforts are being made to develop green synthesis by plants, microbes, and other biological derivatives. The use of plants or plant extracts as reducing and capping or stabilizing agents in the synthesis of nanostructured metals is more useful than other biosynthesis procedures [15]. In addition, cell culture and cell maintenance are not required during the process and the synthesized nanostructure products are more stable, have different shapes and sizes for specific applications, and are inexpensive [16–18]. In recent years, more than thirty plant species have been used for the synthesis of nanostructured CeO_2_, including orange peel, walnut shell, olive leaf, aloe vera leaf, *Prosopis juliflora*, *Jatropha curcas*, and *Elaeagnus angustifolia* [19–25]. These reports suggest that the synthesis of nanostructured CeO_2_ is still a hot topic.

Congo red (CR) is a benzidine-based anionic diazo dye and is widely used in the textile, paper, and plastic industries. It is reported that the theoretical size of CR is 2.29 nm*∗*0.82 nm*∗*0.60 nm based on the Gaussian calculation, which is larger than methylene blue and methylene orange [26]. Another feature of CR is that the size of CR aggregates is up to 40 nm when the concentration is 1 mmol/L [27]. These characteristics make Congo red more difficult to remove compared with other dyes.  Table 1 shows the common technologies used to remove CR such as adsorption, photocatalytic degradation, Fenton-like reaction, and sonocatalytic degradation. Although most of them achieve superior removal efficiency, there are some drawbacks such as high energy requirement and cost for photocatalytic and sonocatalytic degradation and additional chemical reagent requirement for Fenton-like reaction, which lead to a development limitation for the removal of CR. Therefore, adsorption treatment is considered one of the most efficient and convenient methods for removing CR from aqueous solutions.

Tea is one of the most important agricultural products in China and a popular staple food in many Asian countries. Unlike other beverages, most tea is discarded after brewing, causing tea waste to become a serious environmental problem if not treated properly. To solve this problem, many technologies have been developed to reuse tea waste. The most popular use of tea waste is as an adsorbent to remove heavy metals and dyes from aqueous systems. Celesi et al. studied the removal of heavy metal ions (Pb, Cd, Ni, and Zn) using brewed tea waste. The highest efficiency was in the order of Pb (98%)> Cd (85%)>Ni (82%)>Zn (76%) [38]. Nigam et al. showed that the tea waste can remove 71% of total solids, 55% of TDS, and 74.8% of COD from tannery industry wastewater besides significantly high removal of Cr, and the tea waste can be reused up to 5 cycles [39]. In addition to the raw material, the modified tea waste is also widely used. Wen et al. prepared a composite with iron oxide nanoparticle from tea waste for the removal of Cu and Zn, and achieved high adsorption capacities of 95.44 and 68.78 mg/g, respectively [40]. Our studies have shown that the tea waste stem, oolong tea, and black tea have high adsorption capacity of 103.09, 302.63, and 312.50 mg/g for methylene blue, respectively [41–43]. This interpretation suggests that the tea waste and tea-based adsorbents are quite effective in removing pollutants from aqueous systems.

It is well known that tea wastes contain considerable reductive chemical substances such as polyphenols, thearubigins, theaflavins, and theabrownins. These reductive chemical substances are potential candidates for chelation with metals to form a gel-metal precursor, and nanoscale metal oxides are synthesized after the calcination process. From this viewpoint, the reductive chemical substances can be extracted from the tea wastes to be used for the synthesis of nanometals and the remaining tea wastes are further used as adsorbents for the removal of pollutants from the aqueous or gaseous state. This indeed represents a new aspect of tea waste reuse and is an acceptable treatment to create value for tea waste.

As far as we know, the use of the extract obtained from tea waste for the synthesis of nano-CeO_2_ has not been addressed in previous reports. Therefore, the main objective of this study was to prove the feasibility of synthesizing nano-CeO_2_ and evaluate its properties by TGA, XRD, XPS, SEM, and Raman spectroscopy. Moreover, the synthesized CeO_2_ is used to test its ability to adsorb Congo red from aqueous solution and a series of kinetic models, isotherm equations, and thermodynamics are carried out to understand the nature of adsorption. The purpose of this study was to gain a better understanding of the new synthetic method for the preparation of nano-CeO_2_ and its application.

## 2. Experimental

### 2.1. Preparation of Tea Waste and Synthesis Procedure for CeO_2_

Tea waste was collected from the local tea market, Anxi County, Fujian Province. The tea waste samples were pretreated with distilled water to remove unwanted and uncleaned materials present on the surface of the tea waste. The tea waste samples were dried at room temperature for 24 hours and stored in a thermostat. To obtain the extract of tea waste, five grams of tea waste was weighed and mixed with 100 mL of distilled water in a 250 mL beaker. The mixed solution was treated with a 100 rpm for 60 min at 353 K. After cooling, the extract solution was centrifuged at 5000 rpm for 5 min and filtered using a Hirsch funnel to obtain a pure extract of tea waste. To prepare the cerium precursor, 0.05 mol cerium chloride was added to a 20 mL of extract solution and continuously stirred for 12 hours using a magnetic stirrer. After stirring, the gel-cerium precursor was formed and dried at 393 K for 24 hours in an oven. The dried gel-cerium samples were calcined at different temperatures under air atmosphere.

### 2.2. Characteristic of Synthesized CeO2

#### 2.2.1. TGA/DTG Analysis for Weight Loss

Weight loss as a function of temperature for gel-cerium precursor was performed by a thermogravimetric analysis equipped with differential thermal analysis (TGA/DTG, Perkin Elmer Pyris Diamond Model). An amount of 50 mg of gel-cerium precursor was heated in dry air atmosphere to 1073 K with a heating rate of 10°C/min.

#### 2.2.2. X-Ray Diffraction Analysis for Crystalline Structure

The crystalline structures of the synthesized CeO_2_ samples after different calcination processes were determined by X-ray powder diffraction (RIGAKU Model D/MAX III-V) with CuK*α* radiation. The applied current and voltage were 30 mA and 40 kV, respectively. Diffraction patterns were recorded in the angle range 2*θ* = 10–65° at a scan rate of 3°/min.

#### 2.2.3. BET Surface Evaluation

The surface area was measured by adsorption of nitrogen at 77 K using a Micromeritics ASAP 2010 Instrument. Prior to the adsorption measurements, the samples were degassed under vacuum conditions (5 *μ*m Hg) at 373 K for 2 hours. The surface area was calculated by the BET equation.

#### 2.2.4. X-Ray Photoelectron Spectroscopy (XPS)

The surface composition of the synthesized CeO_2_ sample was measured using a VG Micro Lab. MKIII XPS analyzing instrument with an Mg K*α* X-ray radiation source (1253.6 eV). The pressure in the vacuum chamber was maintained at 1.33 × 10^−10^ kPa using an ion pump. The analysis condition was a resolution of 0.1 eV; the number of scans was 100. The binding energy spectra were obtained under the abovementioned conditions and a predetermined scan range.

#### 2.2.5. Scanning Electron Microscopy (SEM) and Transmission Electron Microscopy (TEM)

The morphology of the synthesized CeO_2_ samples was observed by scanning electron microscopy (Philips XL40 FE-SEM). The transmission electron microscopy (TEM) images were performed with a Hitachi 800 with an accelerating voltage of 200 kV.

#### 2.2.6. Raman Spectroscopy

Raman spectra were recorded at room temperature in a wavelength range of 200–1200 cm^−1^ with the spectral resolution of 10 cm^−1^ using a LabRam type Raman spectrometer (Jobin Yvon). A He-Ne laser was used as the exciting source.

### 2.3. Batch Experiment for CR Adsorption

The adsorption activity of the synthesized CeO_2_ for Congo red (CR) was determined in a batch experiment. For comparison, a commercial pure CeO_2_ was experimentally evaluated under identical conditions. All adsorption experiments were carried in a conical flask with a concentration of 100 mg/L and 25 mL of the CR solution. A certain weight of 0.25 g sample and CR solution was placed on a thermal-controlled shaker for 60 min at a sharking rate of 200 rpm. After the adsorption experiment, the solution was centrifuged at 5000 rpm for 10 minutes and the supernatant solution was analyzed to determine the concentration of CR by UV-visible spectrophotometer at 496 nm.

### 2.4. Isotherm and Kinetic Investigations

The Langmuir model is widely used to represent the adsorption of a monolayer that has reacted on the surface of CeO_2_. The Langmuir model can be expressed as follows:

(1) $$\begin{array}{c} \frac{C_{e}}{Q_{e}}=\frac{C_{e}}{Q_{m}}+\frac{1}{Q_{m}K_{L}},\\ R_{L}=\frac{1}{1+C_{0}K_{L}}, \end{array}$$

where *C*_*e*_ and *C*_0_ (mg/L) are the concentration of CR at the equilibrium and initial phases, respectively. *Q*_*m*_ (mg/g) is the maximum adsorption capacity, and *Q*_*e*_ (mg/g) is the adsorption capacity of the equilibrium phase. The dimensionless equilibrium parameter *R*_*L*_ estimates whether the Langmuir isotherm is favorable. When 0 < *R*_*L*_ < 1, the adsorption process is generally considered favorable, while unfavorable is when *R*_*L*_ > 1, as well as *R*_*L*_ = 1 means that the adsorption process is linear; when *R*_*L*_ = 0, the adsorption is irreversible.

The Freundlich model is used to describe the multilayer adsorption of CR molecules on the heterogeneous surface of cerium materials. The equation can be represented as follows:

(2) $$\begin{array}{c} \ln\;Q_{e}=\ln\;K_{F}+\frac{1}{n}\ln\;C_{e}, \end{array}$$

where *K*_*F*_ is the Freundlich constant and *n* is the heterogeneity factor. The value of 1/*n* less than 1 indicates that the interaction between CR molecules and CeO_2_ is favorable.

The Dubinin–Radushkevich model (D-R) is applicable to the adsorption of CR on both homogeneous and heterogeneous surfaces. The linear equation can be represented as follows:

(3) $$\begin{array}{c} \ln\;Q_{e}=\ln\;Q_{D-R}-\beta \varepsilon^{2},\\ \varepsilon =RT\;\ln\left(1+\frac{1}{C_{e}}\right),\\ E=\frac{1}{\sqrt{2\beta }}, \end{array}$$

where *Q*_*e*_ and *Q*_*D-R*_ are the amount of CR adsorbed at equilibrium (mg/g) and the theoretical capacity of CeO_2_ (mg/g). *ɛ* is the Polanyi potential. *β* is related to the mean free energy (*E*) (kJ/mol) of adsorption per mole of CR.

The Temkin model assumes that the heat of adsorption of the molecules in the layer decreases linearly with coverage due to adsorbent-adsorbate interactions and mainly describes the chemisorption process dominated by electrostatic adsorption. The linear form is expressed as follows:

(4) $$\begin{array}{c} Q_{e}=\frac{RT}{B_{T}}\ln\left(A_{T}C_{e}\right), \end{array}$$

where *B*_*T*_ (J/mol) is the Temkin constant and *A*_*T*_ (L/mg) denotes the equilibrium binding constant of the Temkin isotherm.

In this study, the first-order and second-order kinetic models were applied to investigate the adsorption process. The first-order kinetic model was proposed by Lagergren, and it was based on physical adsorption. It is assumed that the adsorption rate is proportional to the change in saturation concentration and adsorption capacities. This linear form of the model can be expressed as follows:

(5) $$\begin{array}{c} \log\left(Q_{e}-Q_{t}\right)=\log\;Q_{e}-\left(\frac{k_{1}}{2.303}\right)t, \end{array}$$

where *Q*_*e*_ and *Q*_*t*_ are the adsorption capacities (mg/g) at the equilibrium phase and at any time *t*, respectively. *K*_1_ (1/min) is the constant rate.

The second-order kinetic model is widely used to illustrate the limiting rate chemical adsorption process. The model is expressed as follows:

(6) $$\begin{array}{c} \frac{t}{Q_{t}}=\frac{1}{k_{2}Q_{e}^{2}}+\frac{t}{Q_{e}}, \end{array}$$

where *Q*_*e*_ and *Q*_*t*_ are the amounts of CR concentration and K_2_ (g/mg/min) is the constant rate of the second-order kinetic model.

## 3. Results and Discussion

### 3.1. TGA/DTG Measurement

**Figure 1:**
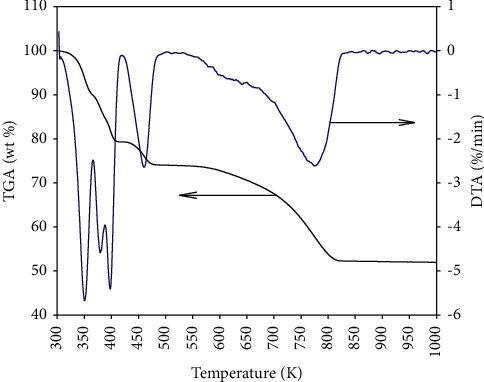
TGA/DTG profile of the gel-phase ceria sample derived from tea extract.

To understand the formation temperature of nanostructure CeO_2_ synthesized from tea extract and cerium precursor, the synthesized sol-gel-cerium sample was measured by TGA/DTG in the airflow and the profile is shown in Figure 1. Four stages of weight loss are observed in Figure 1. The profile of DTG agreed with the result reported by Xue et al. in which their research showed the identical trend and proposed a stepwise dehydration mechanism of CeCl_3_.7H_2_O [44]. They proposed that the dehydration mechanism of CeCl_3_.7H_2_O followed the reaction of CeCl_3_.7H_2_O to CeCl_3_.4H_2_O, and to CeCl_3_.H_2_O and CeCl_3_. The final product was CeO_2_ at a temperature of 863 K. As shown in Figure 1, a large weight loss was found at 773 K, which was attributed to the formation of CeO_2_ through the oxidation reaction of CeCl_3_. Note that the formation temperature of CeO_2_ in this study is much lower than that of Xue et al. [44]. The redox potential of cerium decreases with pH value, which causes the Ce(III) to be easily oxidized to Ce(IV) at the weaker acidity. The pH value of the tea extract used in this study is determined to be ±6.5 providing another favorable driving force for the oxidation of Ce(III) to Ce(IV). In particular, the formation temperature of CeO_2_ can be reached at about 773 K, which reduces the energy consumption and the use of chemical reagents for the synthesis of metal oxides.

### 3.2. XRD Analysis

**Figure 2:**
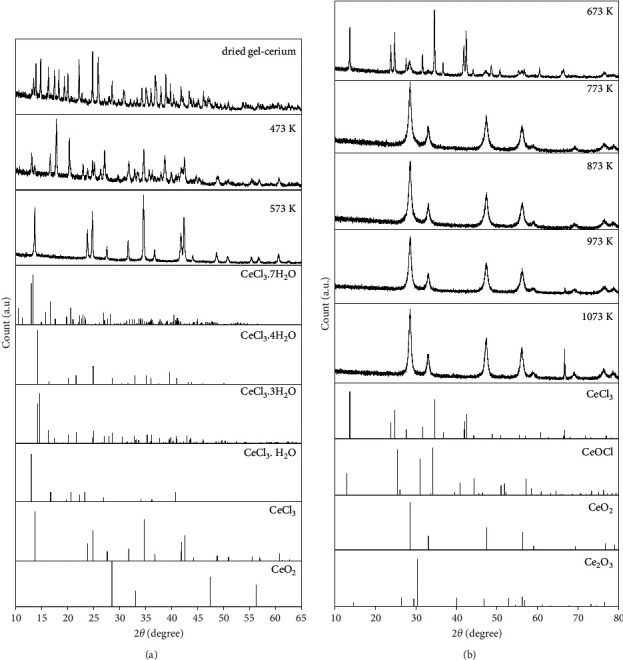
XRD patterns of synthesized Ce calcined at different temperatures (continue).

XRD identification was evaluated to verify the finding and decomposition behavior of gel-cerium from the TGA.  Figure 2 shows the phase transformation of dried gel-cerium sample in the temperature range of 298 K–1073 K. The dried gel-cerium sample shows disordered crystal phases, mainly consisting of CeCl_3_.7H_2_O, CeCl_3_.4H_2_O, and CeCl_3_.3H_2_O, accompanied by unidentified crystal phases. The unidentified crystal phases probably originate chelation and complexation between tea polyphenols and other organic matters during the sol-gel process. The unidentified crystal phases disappear with temperature, and the CeCl_3_ is the main species at 573 K. All the crystal water is decomposed at this stage. It is noteworthy that the tiny crystal peak of CeO_2_ is first detected at 673 K and the well-crystalline CeO_2_ phase is observed at 773 K–1073 K. The 2*θ* values were located at 28.5, 33.1, 47.5, and 56.3 corresponding to the planes of (111), (200), (220), and (311) assigned to the face-centered cubic fluorite structure of CeO_2_ (PFD: 34–0349). This result is corresponding with the TGA measurement, and it is concluded that the formation temperature of CeO_2_ in this study is approximately 773 K. The crystalline size of CeO_2_ at different heat treatments was estimated using the Scherrer equation:

(7) $$\begin{array}{c} D_{\text{scherrer}}=\frac{k\lambda }{\beta \;\cos\;\theta }, \end{array}$$

where *λ* is the X-ray wavelength (0.154 nm), *β* is the full width of the diffraction line at half of the maximum intensity, and *θ* is the half diffraction angle. The calculated results are summarized in Table 2. The crystalline size of CeO_2_ increased with temperature. The crystalline size synthesized in this study ranged from 8.8 to 11.4 nm, which is similar to some synthesis reports [45–48].  Table 3 shows the comparison of CeO_2_ crystal size calculated using the Scherrer equation. As shown, the crystal size of 8.8–11.4 nm is relatively small for all samples, indicating that the tea extract is an acceptable and potential substrate to synthesize the nanostructure CeO_2_. The difference in particle size could be due to the difference in the relaxation of particle surface. In this study, tea polyphenol and large amounts of other substances such as amino acid, theaflavin, and thearubigin are important components of tea extract. During the experimental procedure, the cream-like gel can be easily formed due to the interaction between tea polyphenol, amino acid, theaflavin, and thearubigin. It is believed that the formation of the cream-like gel stabilizes the small particles and reduces the surface relaxation of the forming nanoparticles resulting in smaller lattice parameters. Moreover, note that the lattice parameter decreases with crystalline size and is lower than that reported for CeO_2_ in the standard data JCPDS 34-0349. This result is also in agreement with those of Leoni and Maensiri et al., in which their investigations demonstrated that the lattice parameter of nanocrystalline CeO_2_ is a function of calcination temperature [55, 56]. For nanocrystalline particles, the lattice parameter is found to vary with particle size and can be explained by the relaxation of the grain surface. It is supported that the nanocrystalline particles have a core-shell structure and the core structure is very close to that of bulk monocrystalline cerium oxide. The lattice parameter increases locally at the surface due to the surface trends to relax. In addition, the grain surface relaxation was found to contribute to the line broadening and thus tends to reduce the measured value of dislocation density [57].

### 3.3. SEM and TEM Observation

**Figure 3:**
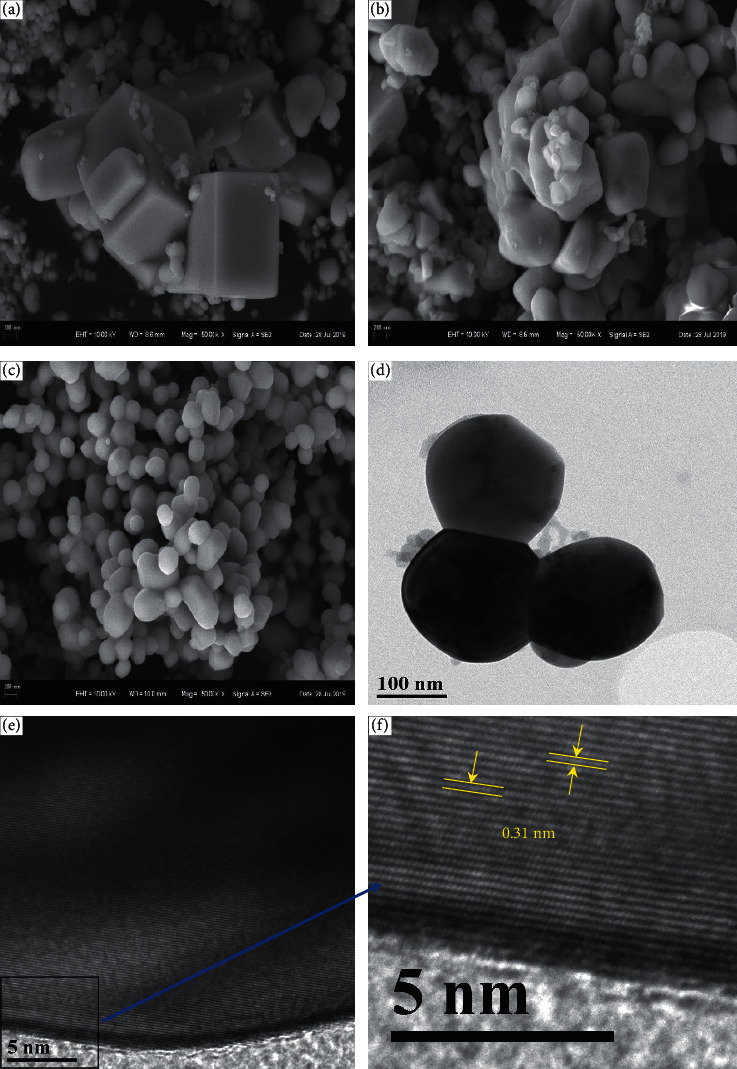
SEM images of synthesized CeO_2_ samples under different heat treatments (a) 723 K, (b) 923 K, and (c) 1073 K, and TEM images of synthesized CeO_2_ at 1073 K (d-f).

Figure 3 shows SEM and TEM images of the synthesized samples obtained at different heat treatments in air atmosphere. A smooth and flat surface of the gel-Ce sample is obviously observed, and the formation of a prism-like shape seems to have been observed at 723 K. Meanwhile, the distinct hexahedral shape of CeO_2_ is formed, which is accompanied by irregular shape and agglomeration. In particular, at 1073 K, a larger amount of homogeneous structure of CeO_2_ with spherical shape and hexahedral trace shape is obtained. It is reported that the morphology, such as crystal size and physiochemical properties of cerium oxide, can be easily controlled using hydroxycarbonates as precursors. The change in shape from prismatic to spherical is attributed to the high content of hydroxycarbonates in tea extract. At high temperatures, CO_3_^2-^ combined with the positively charged groups to form the solid CeCO_3_OH at supersaturation and the phase transformation of CeCO_3_OH to CeO_2_ after heat treatment [58, 59].

The TEM image of synthesized CeO_2_ shows that the sphere has a diameter of about 190 nm. This result is similar to the research report by Zhou et al., in which spherical CeO_2_ crystallites composed of nanoparticles were synthesized by hydrothermal treatment because small CeO_2_ nanoparticles aggregated and gradually evolved into a spherical structure with a low surface energy [60]. As can be seen in Figure 3(f), the value of *d*-space is about 0.31 nm, which corresponds to the (111) lattice plane of CeO_2_. This is in good agreement with the results obtained from XRD and standard data (JCPDS 34-0394). From the SEM observation, the presence of particles is well-defined and the agglomeration property between the particles was also found at the temperature range of 723K–1073 K. Agglomeration is a commonly occurring feature because particles tend to decrease the exposed surface area to lower the surface energy. When two particles are in contact, due to the existence of mismatch between the lattices, the two crystals tend to rotate with each other to minimize the interfacial strain energy. Therefore, the same types of crystal planes tend to align with each other, forming a coherent interface to reduce the interfacial energy [60–62].

### 3.4. XPS Determination

**Figure 4:**
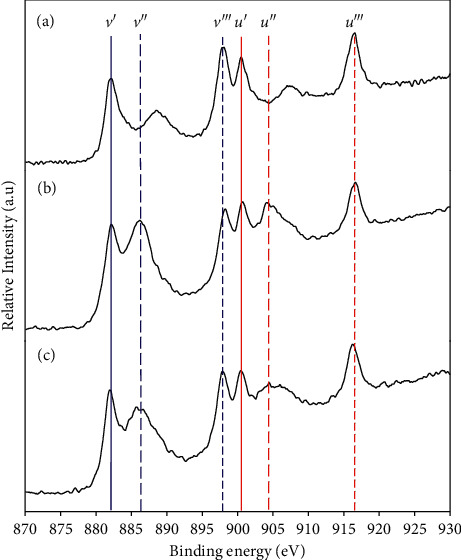
XPS spectra of synthesized CeO_2_ samples calcined at (a) 723 K, (b) 923 K, and (c) 1073 K (v and u indicate Ce 3d5/2 and Ce 3d3/2, respectively).

To examine the chemical state and valence of cerium, the XPS was used to survey the surface composition of the test samples and the spectra of Ce 3d are shown in Figure 4. Typically, the Ce 3d XPS core-level spectra exhibit three spin-orbit doublet features (around 879–890 eV, 895–910 eV, and around 916 eV). In general, it consists of six peaks, corresponding to the pairs of spin-orbit doublets, which can be attributed to the presence of Ce 3*d*_5/2_ and Ce 3*d*_3/2_. As depicted in Figure 4, the labeled *v* and *u* correspond to the structure of Ce 3*d*_5/2_ and Ce 3*d*_3/2_, respectively [63, 64]. The highest binding energy peaks, *v*′′′ and *u*′′′, result from a Ce 3 d^9^ O 2p^6^ Ce 4f^0^ final states. The lowest binding energy peaks, *v*′, *v*′′, *u*′, and *u*′′, are contributed by CeO_2_ and result from a mixture of Ce 3 d^9^ O 2p^5^ Ce 4f^1^ and Ce 3 d^9^ O 2P^4^ Ce 4f^2^ final states. The transfer of electrons from the O 2p to the Ce 4f orbitals and the decrease in the Ce 3d binding energy are due to the interaction of the Ce 4f level with the Ce 3d core hole, which pulls the Ce 4f level to lower energy [65]. Note that the satellite peak *u*′′′ associated with the Ce 3*d*_3/2_ is characteristic of the presence of tetravalent Ce (Ce^4+^ ions) in Ce compounds [63]. For all samples shown in Figure 4, the presence of three spin-orbit doublets (six peaks with satellite peak at 916.5 eV) demonstrated that the Ce (IV) is the predominant species.

### 3.5. Raman Spectra Analysis

**Figure 5:**
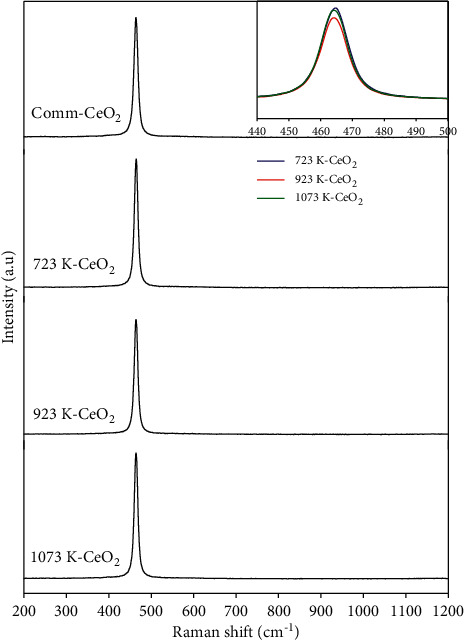
Raman spectra of commercial and synthesized CeO_2_ sample.

Figure 5 shows the Raman spectra of commercial CeO_2_ and synthesized CeO_2_ samples calcined at different temperatures. For all samples, a strong peak located at about 463 cm^−1^ is assigned to the F_2g_ Raman active mode of the cubic fluorite structure of CeO_2_, which is attributed to a symmetric stretching mode of the Ce–O_8_ vibrational unit and the molecule maintains its tetrahedral symmetry throughout [66, 67]. Therefore, this mode should be very sensitive to any disorder in the oxygen sub-lattice resulting from thermal, doping, or grain size-induced effects. Apart from the sharp peak, no other peaks were detected in the Raman spectra. According to the previous investigations, the detectable Raman peaks were measured at about 260 cm^−1^, 570 cm^−1^, 1047 cm^−1^, and 1170 cm^−1^, respectively. The presence of 260 cm^−1^ represents the disorder in the system, while the peak at 570 cm^−1^ is assigned to the defect spaces containing oxygen vacancies [68, 69]. Moreover, the peak centered 570 cm^−1^ can also be probably attributed to the presence of Ce^3+^ [69]. The symmetric characteristic for the synthesized CeO_2_ prepared by calcination at different temperatures appeared to have the same property. No asymmetric characteristic is observed for all samples. It is proposed that, when there is disorder in the oxygen sub-lattice, the Raman active mode is affected by a broadening of the line and an increase in its asymmetry, which is attributed to a decrease in phonon lifetime in the nanocrystalline regime [70, 71]. Based on the XPS and Raman spectroscopic study, it is concluded that the synthesized CeO_2_ derived from the tea extract is more stable and has a relatively ordered structure due to the presence of Ce^4+^.

### 3.6. Adsorption Evaluation for Congo Red (CR)

**Figure 6:**
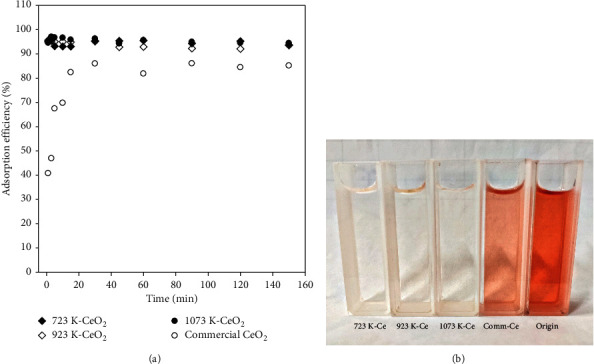
Adsorption efficiency as a function of time for synthesized CeO_2_ samples and commercial CeO_2_ at 298 K with a dosage of 10 g/L, and 100 mg/L of CR (a) performance evaluation and (b) change in color after 1 minute treatment.

For a better understanding of the activity and performance on the adsorption of dye from aqueous solution, a series of synthesized CeO_2_ and a commercial sample were considered for the adsorption of CR due to its carcinogenicity.  Figure 6 shows the adsorption efficiency of CR by a commercial and synthesized CeO_2_ sample calcined at different temperatures. A rapid increase in adsorption efficiency can be found within 5 minutes for all synthesized CeO_2_, implying the excellent reactivity between synthesized CeO_2_ and CR. On the other hand, the adsorption efficiency seems to increase gradually and maintains a steady state for around 30 minutes. This is probably due to the presence of stronger mass transfer resistance between the commercial CeO_2_ and the aqueous CR solution. Note that the color change for all CeO_2_ samples after 1 min treatment shows an extreme difference in Figure 6(b). As shown, the color of CR solution adsorbed by all synthesized CeO_2_ samples derived from tea extract shows a much distinct feature than the commercial CeO_2_, indicating that the adsorption reaction is exactly rapid for the synthesized CeO_2_ samples. The adsorption efficiency is more than 95% for all synthesized CeO_2_ samples, while it is only 41% for a commercial sample when the contact time was set to 1 min. The adsorption efficiency of the synthesized CeO_2_ is more than two times higher than that of the commercial sample. The plausible reasons are (i) the greater surface area of synthesized CeO_2_. The surface area of synthesized CeO_2_ and commercial CeO_2_ is 33.66 m^2^/g and 6.23 m^2^/g, respectively. It is expected that the larger surface area favors the adsorption process and leads to the better adsorption efficiency of CR for synthesized CeO_2_. (ii) The better dispersion of CeO_2_ for the synthesized sample: to understand the dispersion of CeO_2_ on the surface, the CO_2_ temperature-programmed desorption (CO_2_-TPD) was employed. The desorbed amount of CO_2_ at 400°C for synthesized CeO_2_ was estimated to be 61.6 *μ*mol-CO_2_/g, while it was 12.5*μ*mol-CO_2_/g for commercial CeO_2_. This result is an important finding that supports the adsorption experiment for the removal of CR. The larger desorbed amount of CO_2_ indicates a better dispersion of CeO_2_ on the surface. From the above results, it can be concluded that the synthesized CeO_2_ from tea waste has better adsorption behavior, which can be contributed to the better dispersion of CeO_2_ on the surface compared with the commercial CeO_2_.

### 3.7. Effect of Initial CR Concentration

**Figure 7:**
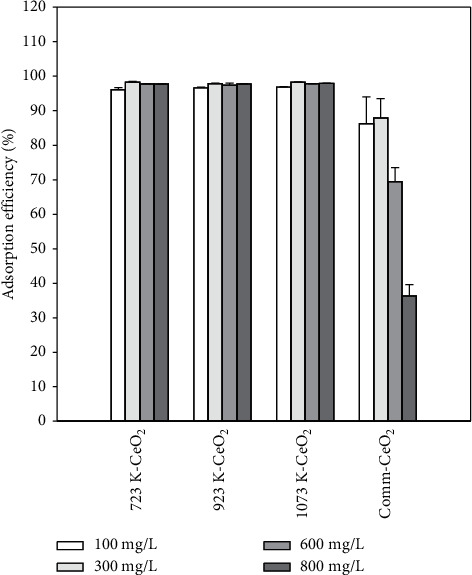
Adsorption efficiency as a function of CR concentration for synthesized CeO_2_ samples and commercial CeO_2_ at 298 K with a dosage of 10 g/L and contact time of 60 min.

The effect of the initial CR concentration on the adsorption of CR at 298 K with a dosage of 10 g/L and a contact time of 60 min was investigated. As can be seen in Figure 7, the adsorption efficiency of all synthesized CeO_2_ samples maintains a steady state in the range of 96%–98% and no deactivation feature is observed within the experimental conditions. The adsorption efficiency is as high as approximately 98% even at 800 mg/L. For a commercial CeO_2_, the adsorption efficiency is more than 85% when the CR concentration is between 100 and 300 mg/L and decreases to 70% at 600 mg/L, while the worst performance is observed at 800 mg/L. Based on the experimental results, it is suggested that the synthesized CeO_2_ has a greater chemical affinity with CR than the commercial sample and is likely associated with multilayer adsorption mechanism with CR. Moreover, the synthesized CeO_2_ samples showed better adsorption efficiency, which is attributed to the uniform dispersion of CeO_2_ active sites throughout the bulk sample, thus promoting the overall adsorption efficiency even when the CR concentration was set to 800 mg/L. The ANOVA shows that the *p* value is much smaller than 0.01 (*p* value is 5.21*∗*10^−8^) for the concentration of CR, implying very significant differences between the adsorption efficiency and the concentration of CR.

### 3.8. Effect of Reaction Temperature

**Figure 8:**
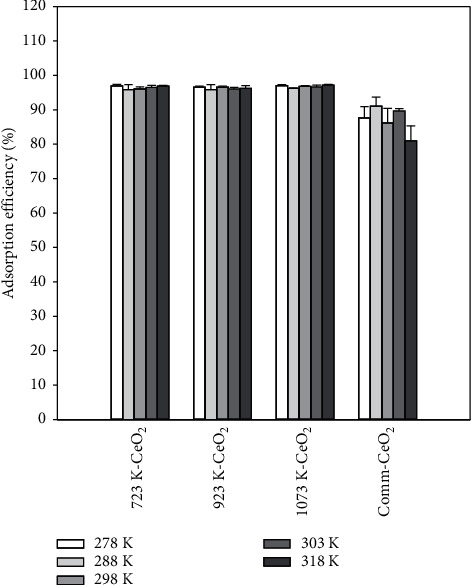
Adsorption efficiency as a function of temperature for synthesized CeO_2_ samples and commercial CeO_2_ at a dosage of 10 g/L, 100 mg/L of CR, and contact time of 60 min.

The effect of temperature in the range of 278 K–318 K on the adsorption of CR with a dosage of 10 g/L and a contact time of 60 min was investigated. As shown in Figure 8, it was found that the adsorption efficiency maintains about 97% and does not change significantly in the range of 278 K–318 K for all synthesized CeO_2_, which can be explained by the fact that temperature is not a key factor to govern the overall reaction. The heat of reaction between the synthesized CeO_2_ and CR would reach the steady state under the experimental conditions. Unlike synthesized CeO_2_, the adsorption efficiency of commercial CeO_2_ decreased gradually with temperature, which can be explained by the fact that higher temperature may cause the desorption rate to be more favorable than that of adsorption, resulting in the release of part of the adsorbed CR from the solid phase to the liquid phase, thus decreasing the adsorption efficiency. Based on the experimental results, it is believed that the synthesized CeO_2_ can be used in the temperature range of 278 K–318 K under the experimental conditions in this study. The ANOVA shows that the *p* value is much smaller than 0.01 (*p* value is 7.54*∗*10^−11^) for the reaction temperature, implying very significant differences between the adsorption efficiency and the reaction temperature in this study.

### 3.9. Study of Adsorption Kinetic

Adsorption kinetics provides valuable information on the adsorption rate and relevant coefficients for pilot scale and engineering design. The first-order and second-order kinetic models were applied to fit the adsorption reaction and to investigate the nature of the process. The fitting results of the two models are summarized in Table 4. The correlation coefficient values (*R*^2^) of the second-order model for synthesized and commercial CeO_2_ are much better than those of the first-order model. Meanwhile, the fitted adsorption capacities (*Q*_fit_) of synthesized and commercial CeO_2_ derived from the second-order model are estimated to be 9.5 mg/g and 4.07 mg/g, which is close to the experimental result (*Q*_exp_) in this study. These results support that the second-order kinetic model is more suitable for understanding the adsorption process between CeO_2_ and CR. The well-fitting result for second-order model demonstrates that the rate-limiting step is not the resistance of the boundary layer [72]. The adsorption process is controlled by chemisorption, which involves valence forces due to the sharing or exchange of electrons between the adsorbent and the adsorbate [73, 74]. The second-order model includes the external liquid film diffusion, intraparticle diffusion, and adsorption on the surface of the adsorbent; this model provides a more comprehensive and accurate description of the adsorption mechanism between powder CeO_2_ and CR [75, 76].

### 3.10. Adsorption Isotherm Models

The fitting results and parameters of four isotherm models are listed in Table 5. For the synthesized CeO_2_, with the exception of the Langmuir model, the higher *R*^2^ values were found in the range of 0.93–0.97 for other models. This suggested that the Langmuir model is not suitable to describe the interaction between synthesized CeO_2_ and CR. The Freundlich model shows the best fitting result among all the models, indicating that the multilayer adsorption is the main mechanism, leading to rapid reaction and greater adsorption efficiency within the short contact time. However, the high value of *R*^2^ derived from the Langmuir model for the commercial CeO_2_ indicated that the interaction between the commercial CeO_2_ and CR may be attributed to the monolayer adsorption, thus leading to the gradual increase in adsorption efficiency with contact time. In addition, the D-R model shows the superior *R*^2^ values for both synthesized CeO_2_ and commercial CeO_2_. The mean free energy (*E*) derived from D-R model was 0.32 kJ/mol and 0.11 kJ/mol for the synthesized and commercial CeO_2_, respectively, which is classified as the physisorption interaction. Moreover, the Temkin model also shows the high *R*^2^ value for both synthesized CeO_2_ and commercial CeO_2_, which implies that the electrostatic interaction probably is one of the mechanisms between CeO_2_ and CR. By comparing the values of *R*^2^ of the examined four isotherm models, it is concluded that the D-R and Temkin models fit well for both synthesized CeO_2_ and commercial CeO_2_, and the Langmuir model is suitable for commercial CeO_2_, while the Freundlich model is the best model for the synthesized CeO_2_ in this study.

### 3.11. Thermodynamics Investigation

To determine the thermodynamic parameters for the adsorption of CR by synthesized CeO_2_, experiments were conducted at 278 K, 288 K, 298 K, 303 K, and 318 K using a 100 mg/L of CR solution. The Gibbs free energy, *ΔH*^0^, and *ΔS*^0^ were calculated, and the expression is as follows:

(8) $$\begin{array}{c} \Delta G^{0}=-RT\;\ln\;k,\\ \ln\;k=\frac{\Delta S^{0}}{R}-\frac{\Delta H^{0}}{RT}, \end{array}$$

where *k* is the rate constant related to the equilibrium phase. *ΔH*^0^ and *ΔS*^0^ are the enthalpy and entropy, respectively. *T* (K) is the absolute temperature, and *R* (8.314 J/mol/K) is the gas constant. The calculated parameters are shown in Table 6. The ΔG values are −7.49, −7.68, −8.31, −7.93, and −8.83, respectively, indicating that the adsorption of CR by synthesized CeO_2_ is a spontaneous reaction. The values of *ΔH*^0^ and *ΔS*^0^ are 0.002 kJ/mol and 0.032 kJ/mol/K, respectively. Therefore, the adsorption process can be assumed to be an endothermic reaction with the increase in the degree of disorder. The analysis of thermodynamics is similar to the fitting results of kinetic model.

### 3.12. Comparison of Other Adsorbents for CR Removal

The comparison of the maximum adsorption capacity of CeO_2_ for CR with that of other adsorbents in the literature is shown in Table 7. The CeO_2_ samples show comparable adsorption capacity for CR as compared to the adsorbents. The synthesized CeO_2_ sample has relatively high adsorption capacity among the adsorbents. Therefore, synthesized CeO_2_ is a suitable adsorbent for the removal of CR.

## 4. Conclusion

This study reported the synthesis of nanostructure CeO_2_ powder using extract obtained from tea waste. The synthesized CeO_2_ samples were characterized by thermogravimetric analyzer (TGA), X-ray diffraction (XRD), scanning electron microscopy (SEM), X-ray photoelectron spectroscopy (XPS), and Raman spectroscopy.

From the TGA/DTG analysis, the intermediate of cerium chloride hydrates such as CeCl_3_.4H_2_O and CeCl_3_.H_2_O and CeCl_3_ was produced and it was proposed that the formation temperature of CeO_2_ was estimated to be 773−K. The cubic fluorite structure of CeO_2_ was detected to be the predominant species and was completely formed at the calcination temperature of 773 K–1073 K with a crystal size between 8.8 and 11.4 nm based on the XRD measurement. Moreover, the main chemical state of ceria on the surface of the synthesized samples was confirmed to be tetravalent ceria by XPS. All the samples show a strong Raman signal at a well-defined chemical shift at about 463 cm^−1^ and a significant symmetry feature was observed, inferring that the tetravalent ceria is the dominant species throughout the bulk sample. Except for commercial CeO_2_, a rapid increase in adsorption efficiency for all synthesized samples indicated better reactivity between synthesized CeO_2_ and CR. The larger surface area and uniform dispersion of CeO_2_ on the surface could be the main reason for the difference in adsorption efficiency of synthesized and commercial samples. For all synthesized CeO_2_ samples, there is no difference in adsorption efficiency even when the concentration of CR was controlled to 800 mg/L. The adsorption efficiency was still more than 95%, while a distinct decrease was observed in the commercial sample. The same trend is reflected in the effect of reaction temperature. From the kinetic analysis and isothermal fitting, the second-order model was found to be the appropriate model to interpret the adsorption behavior of synthesized CeO_2_. The calculated adsorption capacity derived from the second-order model agreed well with the experimental data. The isothermal fitting results indicated that the Langmuir model is suitable for commercial CeO_2_, where the adsorption mechanism is dominated by a monolayer, while the Freundlich model is the best model for the synthesized CeO_2_ in this study. This also provided a useful explanation that the synthesized CeO_2_ is better than the commercial sample as multiple layer adsorption of CR is possible for synthesized CeO_2_. Thermodynamic investigation indicated that the adsorption mechanism between synthesized CeO_2_ and CR was a spontaneous and endothermic process with the increase in the degree of disorder. In conclusion, the extract of tea waste is a readily available and environmentally friendly raw material for the synthesis of nanostructure CeO_2_.

## Figures and Tables

**Table 1:** The common technologies used to remove Congo red.

Material	Year	*Q*_max_ (mg/g) removal efficiency	Degradation mechanism	Reference
Coir pith carbon	2002	6.72	Adsorption	[28]
Na-Bentonite	2009	35.8	Adsorption	[29]
Hollow zinc ferrite	2011	16.58	Adsorption	[30]
Cellulose/PVC/ZnO	2016	90%	Photocatalytic	[31]
PdZnO-3	2016	98.2%	Photocatalytic	[32]
Fe^0^/PANI	2017	98%	Sonocatalytic degradation	[33]
ZnO-ES	2018	91.6%	Photocatalytic degradation	[34]
Fe_2_O_3_@CeO_2_–ZrO_2_	2018	95%	Fenton reaction	[35]
Desiccated coconut	2021	49.46	Adsorption	[36]
Cu–Ca–Al-layered double hydroxide	2021	81	Adsorption	[37]

**Table 2:** The calculated *d*-spacing, crystalline size, and lattice parameter of synthesized CeO_2_ after different heat treatments.

Calcination temperature (K)	*d*-spacing (nm)	Crystalline size (nm)	Lattice parameter (nm)
773	0.3123	8.8 ± 0.12	0.5408
873	0.3121	9.8 ± 0.14	0.5406
973	0.3121	10.1 ± 0.13	0.5405
1073	0.3121	11.4 ± 0.16	0.5406

**Table 3:** Comparison of CeO_2_ crystal size derived from XRD calculation.

Materials	Substrate	Crystal size (nm)	Reference
Mn-doped CeO_2_	CeCl_3_·H_2_O	7.9–16.64	[49]
CeO_2_@C	Ce(NO_3_)_3_·6H_2_O	11.14–12.12	[50]
CeO_2_, Ce_1-x_Ag_x_O_2_	Ce(NO_3_)_3_·6H_2_O	18.8–140.9	[51]
CeO_2_	Ce(NO_3_)_3_	20–26.2	[52]
CeO_2_	Ce(NO_3_)_3_·6H_2_O	10	[53]
CeO_2_	Ce(NO_3_)_3_	30–45	[54]
CeO_2_	CeCl_3_·7H_2_O	12.53–16.07	[47]
CeO_2_	CeCl_3_·7H_2_O	8.8–11.4	This study

**Table 4:** Fitting results for the first-order and second-order kinetic models.

		First-order model			Second-order model		
Sample	*Q*_exp_ (mg/g)	*k*_1_ (1/min)	*R* ^2^	Q_fit_ (mg/g)	*k*_2_ (1/min)	*R* ^2^	*Q*_fit_ (mg/g)
873K–CeO_2_	9.55 ± 0.21	0.02	0.44	41.66 ± 1.23	0.11	0.99	9.50 ± 0.54
Comm-CeO_2_	8.61 ± 0.17	0.03	0.37	30.30 ± 0.87	0.25	0.99	4.07 ± 0.22

**Table 5:** Adsorption parameters obtained from the Langmuir, Freundlich, D-R, and Temkin isotherm models.

		873K–CeO_2_	Comm-CeO_2_
Langmuir	*K*_L_ (L/mg)	0.03	0.17
	*R*_L_	0.04–0.27	0.01–0.06
	*R* ^2^	0.34	0.98
Freundlich	1/*n*	0.45	0.06
	*K*_F_	2.61	5.80
	*R* ^2^	0.97	0.60
D-R	K(mol^2^/kJ^2^)	4.92	44.22
	E(kJ/mol)	0.32	0.11
	*R* ^2^	0.96	0.95
Temkin	*B*_T_ (J/mol)	78.65	124.21
	*A*_T_ (L/mg)	8.37	6.13
	*R* ^2^	0.93	0.95

**Table 6:** Calculated thermodynamic parameters derived from the Van't Hoff equation at different temperatures.

Temperature (K)	△G^0^ (kJ/mol)	△H^0^ (kJ/mol)	△S^0^ (J/mol/K)
288	-7.49 ± 0.24	0.002 ± 7.8^*∗*^10^−5^	0.032 ± 1.2^*∗*^10^–3^
298	-7.68 ± 0.31		
308	-8.31 ± 0.32		
318	-7.93 ± 0.38		
323	-8.83 ± 0.47		

**Table 7:** Adsorption capacity of CR on different adsorbents.

Adsorbent	BET (m^2^/g)	Adsorption capacity of CR (mg/g)	Reference
Activated red mud	20.70	7.08	[77]
Activated coir pitch	—	6.72	[28]
Kaolin	20.28	5.44	[78]
Zeolite	8.31	3.77	[78]
Anilinepropylsilica xerogel	150	22.62	[79]
Commercial CeO_2_	6.23	4.07	This study
Synthesized CeO_2_	33.66	9.50	This study

## Data Availability

The data generated and analyzed in this manuscript are available from the corresponding author on reasonable request.
